# Predicting Treatment Response in Depression: The Role of Anterior Cingulate Cortex

**DOI:** 10.1093/ijnp/pyy069

**Published:** 2018-08-16

**Authors:** Beata R Godlewska, Michael Browning, Ray Norbury, Artemis Igoumenou, Philip J Cowen, Catherine J Harmer

**Affiliations:** 1 Psychopharmacology Research Unit, University Department of Psychiatry, University of Oxford, Oxford, United Kingdom; 2 Oxford Health NHS Foundation Trust, Warneford Hospital, Warneford Lane, Oxford, United Kingdom; 3 Computational Psychiatry Lab, University Department of Psychiatry, University of Oxford, Oxford, United Kingdom; 4 Department of Psychology, Whitelands College, University of Roehampton, London, United Kingdom; 5 Barnet Enfield and Haringey Mental Health NHS Trust, United Kingdom; 6 Psychopharmacology and Emotion Research Laboratory, University Department of Psychiatry, University of Oxford, Oxford, United Kingdom

**Keywords:** anterior cingulate cortex, depression, selective serotonin reuptake inhibitors, treatment response prediction, functional magnetic resonance imaging

## Abstract

**Background:**

Identification of biomarkers predicting therapeutic outcome of antidepressant treatment is one of the most important tasks in current research because it may transform the lengthy process of finding the right treatment for a given individual with depression. In the current study, we explored the potential of pretreatment pregenual anterior cingulate cortex activity as a putative biomarker of treatment response.

**Methods:**

Thirty-two medication-free patients with depression were treated for 6 weeks with a selective serotonin reuptake inhibitor, escitalopram. Before treatment began, patients underwent an fMRI scan testing response to brief, masked, presentations of facial expression depicting sadness and happiness.

**Results:**

After 6 weeks of treatment, there were 20 selective serotonin reuptake inhibitor responders and 12 nonresponders. Increased pretreatment pregenual anterior cingulate cortex activity to sad vs happy faces was observed in responders relative to nonresponders. A leave-one-out analysis suggested that activity in the anterior cingulate cortex was able to predict response status at the level of the individual participant.

**Conclusions:**

The study supports the notion of pregenual anterior cingulate cortex as a promising predictor of antidepressant response.

**Significance Statement**

Most patients with depression do not respond to the first treatment they try, and in many cases multiple attempts are necessary to relieve symptoms. The fact that depression affects about 20% of the population at least once in their lifetime means that this problem applies to a substantial number of individuals. The delay in finding effective treatment, often extending to months or years, translates into unnecessary personal suffering and burden. This burden could be lessened if there were tests indicating those patients who are unlikely to respond to first-line treatment and require higher intensity treatment from outset. This study, using functional imaging, has added to the growing body of evidence pointing at the pregenual anterior cingulate cortex as a reliable predictor of subsequent treatment response in depression. Such studies bring us closer to the application of biological markers to predict therapeutic response in clinical practice.

## Introduction

The search for biomarkers that can predict clinical response to the pharmacotherapy of depression is a task of substantial practical importance. Only 50% of patients respond to the first treatment they try, and remission rates are even lower (around 30%) (Rush et al., 2009). Many patients will take 2 or more different antidepressants before finding a drug that works for them (Warden et al., 2007). Identifying patients unlikely to respond to first-line treatment may speed up the application of second-line or adjunct treatments and improve overall time to remission.

Studies employing neuroimaging have led to the identification of a number of candidates for treatment response prediction biomarkers (e.g., McGrath et al., 2013; Dunlop et al. 2017). One of the best established is increased pretreatment activity in the pregenual anterior cingulate cortex (pgACC), which has been linked to a positive therapeutic response to antidepressant treatments. In a review of 23 imaging studies, Pizzagalli (2011) concluded that increased activity in the rostral anterior cingulate cortex (ACC) (equivalent to pgACC) both in the resting state and in response to simple cognitive tasks was associated with positive outcome to a variety of treatment modalities, including pharmacotherapy, transcranial magnetic stimulation, and sleep deprivation. This was also shown in a meta-analysis by Fu et al. (2013).

Of the studies reviewed by Pizzagalli (2011) and Fu (2013), 5 and 14, respectively, employed fMRI, the most widely available modality for imaging the ACC in depressed patients. Since these publications, a number of further fMRI studies have been conducted, including a study by our group in which we reported that neural changes in response to an emotional processing task after 1 week of treatment with the selective serotonin reuptake inhibitor (SSRI) escitalopram predicted clinical outcome after 6 weeks of treatment in a group of 32 depressed patients (Godlewska et al., 2016). The present report concerns baseline (pretreatment) neural responses of this patient group to a different emotional task that employed masked faces as “implicit” (nonconscious) stimuli and their potential to act as predictors of subsequent response to escitalopram.

Interestingly, a similar implicit task was employed in a recent fMRI investigation, which also found a correlation between pretreatment activity in pgACC during the emotional processing task and the subsequent response to 8 weeks of treatment with the SSRI sertraline in 10 unmedicated patients (Victor et al. 2013). This study was designed to assess whether the results can be replicated in a larger group, allowing for a categorical classification of patients into responders and nonresponders. A similar task was used based on the concept that structures involved in rapid, nonconscious stimulus processing may be particularly reactive to masked stimuli and sensitive to depression (Victor et al. 2010, 2013, 2017).

The aim of the present study was to test this hypothesis that increased pgACC to masked sad facial expressions at baseline would predict later treatment response and provide an initial estimate of the degree to which this effect was able to predict treatment response at the level of the individual patient, using a leave-one-out (LOO) validation process.

## Methods

### Participants

Thirty-nine patients with major depression consented to take part in the study. Thirty-two (18F:14M) completed the fMRI scan and the 6-week period of escitalopram treatment (see Table 1 for demographic information). In the remaining 7 patients, relevant data were not available at the end of the treatment period (4 patients dropped out before the 6-week assessment, and scanning data from a further 3 patients were not available due to technical issues). All participants were assessed with the Structured Clinical Interview for DSM-IV (Spitzer et al., 1995) for the presence of current and past psychiatric disorders. The depressed patients met criteria for a primary diagnosis of major depressive disorder; exclusion criteria were psychosis or substance dependence as defined by DSM-IV, a clinically significant risk of suicidal behavior, contraindications to escitalopram treatment or treatment with psychotropic medication <3 weeks before the study (5 weeks for fluoxetine), major somatic or neurological disorders, pregnancy or breast-feeding, any contra-indications to MR imaging, or concurrent medication that could alter emotional processing. All participants were right-handed. The study was approved by the Oxford Research Ethics Committee and all participants gave written informed consent.

**Table 1.** Demographic Information for Responders and Nonresponders to 6 Weeks Treatment with Escitalopram

	Responder (n=20)	Nonresponders (n=12)	
Gender	10F/10M	8F/4M	*P*=.358, χ2=0.847
Age at time of scan (years)	28.25±2.64	28.75±9.28	*P*=.886, t=0.144
Baseline depression severity (HAM-D)	23.0±1.1	23.67±0.9	*P*=.687, t=0.407
Baseline depression severity (BDI-I)	31.2±1.5	33.1±1.5	*P*=.420, t=0.818
Baseline trait anxiety (STAI-T)	59.5±1.9	63.2±10.8	*P*=.298, t=1.059
Duration of current episode (months)	4.5±0.6	8.8±2.6	*P*=.06, t=1.992

Abbreviations: HAM-D, Hamilton Depression Rating Scale; BDI-I - Beck Depression Inventory I; STAI-T, Spielberger’s State-Trait Anxiety inventory; F, females; M, males.

Presented as mean±SE.

### Study Design and Drug Treatment

Following the baseline fMRI scan, patients received 10 mg escitalopram each morning for a period of 6 weeks without dose adjustment. Assessment of depressive severity and treatment response was made using the 17-item Hamilton Depression Rating Scale (HAM-D) (Hamilton, 1960), with anxiety being measured with Spielberger’s State-Trait Anxiety inventory (Spielberger, 1989) at baseline and week 6. The fMRI assessments were completed at the same time points. The current analysis focuses on how baseline differences in the function of pgACC were able to predict clinical response at week 6 of treatment. After the 6-week duration of the study, all patients were offered treatment openly with escitalopram according to usual clinical practice. Clinical response to the SSRI was defined as a reduction in HAM-D of 50% or more from baseline after 6 weeks of treatment (Angst et al., 1993).

### fMRI Data Acquisition

fMRI data were acquired on a 3T Siemens TIM TRIO (Siemens AG). Data were acquired with a voxel resolution of 3×3×3mm, TR/TE/FA=2000 milliseconds/28 milliseconds/89^o^. A total of 256 volumes were acquired in an experiment lasting 6 minutes. T_1_-weighted structural images were acquired using a magnetization prepared rapid acquisition by gradient echo sequence with a voxel resolution 1.0×1.0×1.0 mm on a 208×256×200 grid, TE/TI/TR=4.68/900/2040 magnetization prepared rapid acquisition by gradient echo sequence. To monitor cardiac and respiratory processes, subjects wore a pulse oximeter and respiratory bellows.

### fMRI Experimental Task

During fMRI scanning, participants completed a backward masking task. This task consisted of viewing pairs of faces paired in such a way that the first face, expressing sad, happy, or neutral emotion, was shown for 30 milliseconds and then immediately “masked” by another face of neutral expression, shown for 70 milliseconds; this procedure has been shown to interfere with explicit perception of the first face (Victor et al. 2010). After each pair of faces was presented for 100 milliseconds in total, participants were asked to report the gender of the face via an MRI compatible key pad; the gender of both faces was the same. Each participant was shown 4 sad blocks, 4 happy blocks, and 9 neutral blocks, which were interleaved with sad and happy blocks (N-S-N-H-N-S-N-H-N or N-H-N-S-N-H-N-S-N). Between each block, there was a 10-second block of a baseline fixation cross.

### fMRI Preprocessing and Statistical Analysis

Functional MRI data were preprocessed and analyzed using FMRIB software library (FSL) (Jenkinson et al. 2012). Briefly, motion correction was applied using a rigid body registration to the central volume; brain matter was segmented from nonbrain using a mesh deformation approach. Gaussian spatial smoothing was applied with a full-width half maximum of 5 mm; high-pass temporal filtering was applied using a Gaussian-weighted running lines filter, with a 3-dB cut-off of 120 seconds.

A general linear model was fitted in prewhitened data space. Three explanatory variables (plus their temporal derivatives) were modelled: sad faces, happy faces, and neutral faces. All explanatory variables were convolved with a default haemodynamic response function (Gamma function, delay=6 seconds, standard deviation=3 seconds)and filtered by the same high-pass filter as the data. The impact of physiological noise on the BOLD signal was reduced using the Physiological Noise Modelling tool of FSL. Pulse oximetry and respiratory bellows data were processed by Physiological Noise Modelling to create 33 nuisance regressors, which were added to the first-level fMRI model. The full model was simultaneously regressed against the BOLD data, giving the best-fitting amplitudes for each explanatory variable while accounting for the physiological noise.

The task contrast of interest in this study was the relative activation of sad vs happy faces. The degree to which the change in neural activity in this contrast predicted participants’ clinical response on the HAM-D to medication over 6 weeks was tested using a 2-level analysis. The first level consisted of the sad vs happy contrast maps, as described above, calculated for each depressed subject. Second-level, between-subject, random effects analysis assessed whether this change in neural activity differed between depressed patients who went on to respond to the medication and those who did not. Baseline HAM-D score was included as a regressor in the second-level analysis to account for the potential influence of initial depression severity effects on overall clinical response (NB baseline HAM-D score did not differ between the 2 groups, responders and nonresponders, Table 1, and equivalent results were obtained when the analysis was run without this covariate; supplemental Table 4).

The mask for the ACC as a priori region of interest (ROI) was derived from the Harvard-Oxford Cortical Anatomical Atlas and used in small volume correction analysis (SVC; clusters determined by Z>2.3 and a (corrected) cluster significance threshold of *P*=.05). The mask included 1531 voxels. The results of group-level whole-brain analyses were corrected using cluster-based thresholding with a height threshold of Z>2·3 and a (whole-brain corrected) spatial extent threshold of *P*=.05.

Additional analysis was performed with randomized, FSL’s nonparametric tool using Threshold-Free Cluster Enhancement thresholding approach with baseline HAMD as a nuisance variable. This allowed for nonparametric permutation-based inference without a predefined arbitrary threshold, reducing the likelihood of false positive results. 5.000 permutations were performed.

### Predictive Analysis

In addition to the analysis described above in which participant groups were defined based on future response to treatment, we were also interested in whether activity of the ACC could be used to predict response for individual patients. This was done using a LOO approach in which training and testing data were kept completely separate. This analysis involved firstly defining a cluster of voxels in which activity was greater for responders than nonresponders to the sad-happy contrast within the ACC (NB the cluster was defined using Z>2.3 and *P*<.05 with a structural ROI based on the Harvard-Oxford Cortical Atlas) using just the training data (i.e., data from all but 1 participant). Secondly, mean activity was extracted within this cluster for all participants and the data from the training sample used to generate a receiver operator characteristic curve. Lastly, a cut-off was defined from the receiver operator characteristic curve of the training data as the point furthest from the leading diagonal and the held-out participant classified as a responder or nonresponder based on this cut-off. Note that this analysis was repeated for every participant and resulted in different clusters of voxels used in each classification as well as different cut-offs for the classification. This analysis provides an initial estimate of the ability of activity within the ACC to predict response at the level of the individual patient.

## Results

### Clinical and Demographic Data

After 6 weeks’ escitalopram treatment, 20 of 32 patients (62%) were classified as responders. There were no differences between responders and nonresponders in terms of gender, age, baseline depression severity, baseline trait anxiety, or duration of current episode (Table 1).

### fMRI Data

**Figure 1.**
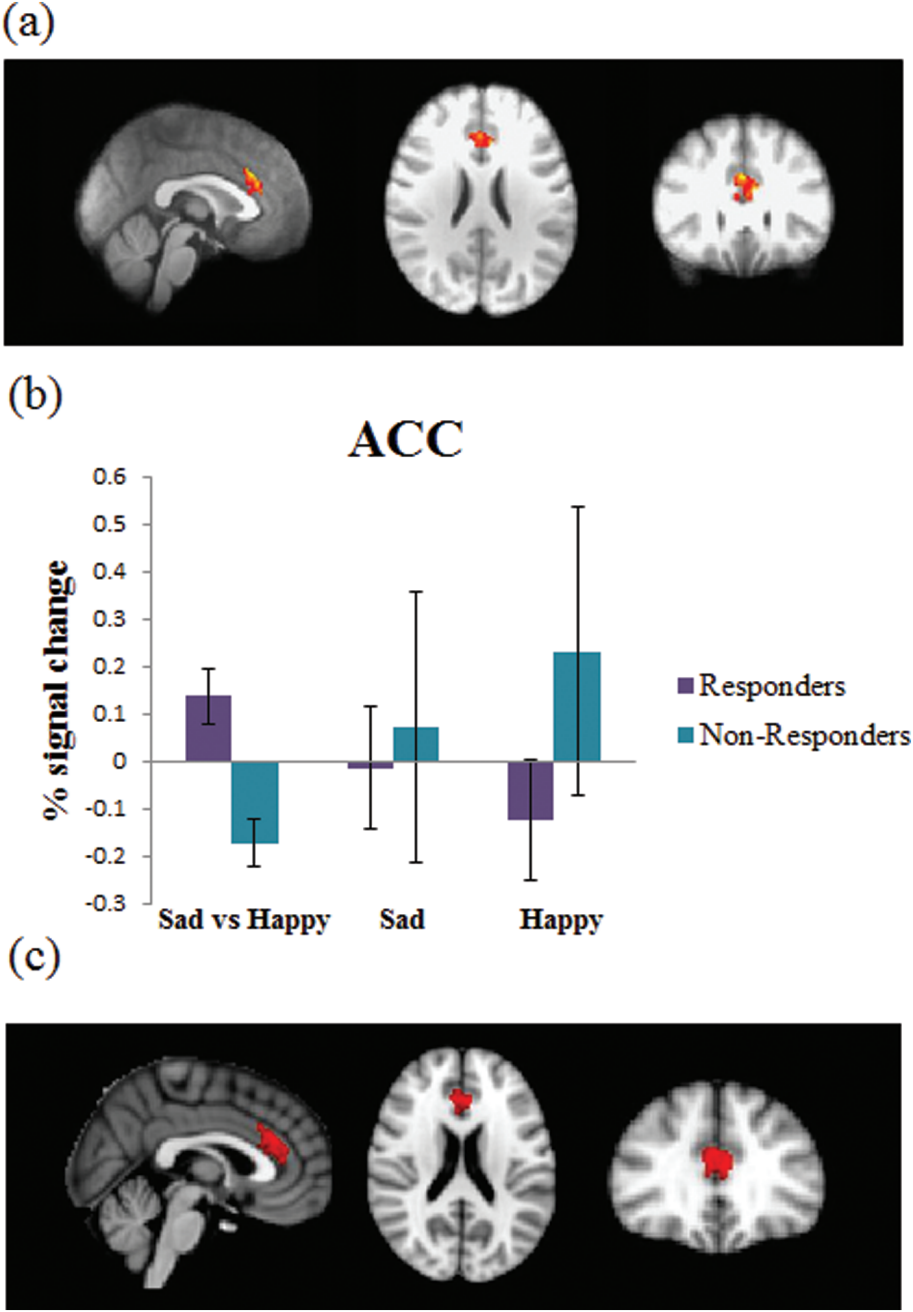
Baseline differences in neural response (percent signal change) in the pregenual anterior cingulate cortex (pgACC) region of interest (ROI) in response to sad vs happy facial expressions differentiated between responders and nonresponders to 6 weeks of treatment with escitalopram. The figure represents (a) results of small volume correction (SVC) analysis in the anterior cingulate cortex (ACC) using a parametric approach (Gaussian Random Field Theory); (b) extracted signal change in the identified cluster (mean and standard error); (c) results of SVC-corrected analysis in the anterior cingulate cortex using a nonparametric approach (Threshold-Free Cluster Enhancement). Analysis was thresholded at Z=2.3 and cluster-corrected with a family wise error (FWE) *P*<.05. Baseline 17-item Hamilton Depression Rating Scale (HAM-D) scores were entered as a covariate.

**ROI Analysis—**We performed SVC-corrected analysis of pgACC. In line with our hypothesis, we found an increased fMRI response to sad vs happy faces in the group of patients who after 6 weeks responded to treatment compared with those who did not, both with and without controlling for baseline depression HAMD severity (family wise error [few]-corrected *P*<.05, SVC; 248 voxels, Z-value of the peak voxel 3.48, *P*=.005). Exploring each emotion separately vs baseline showed that nonresponders had numerically greater pgACC responses to both sad and happy faces, with the majority of the difference between the 2 groups apparently being due to altered responses to happy faces (Figure 1).

While we used an ACC mask based on the commonly used Harvard-Oxford Cortical Anatomical Atlas, the part of the cluster identified can be described as located in the anterior midcingulate cortex. An additional analysis with a 600-voxel mask consisting only of volumes anterior to the genu of corpus callosum was run, and a similar response to that reported above was observed in a cluster of 123 voxels, the part of the original cluster corresponding to strictly defined ACC (results not reported).

**Figure 2.**
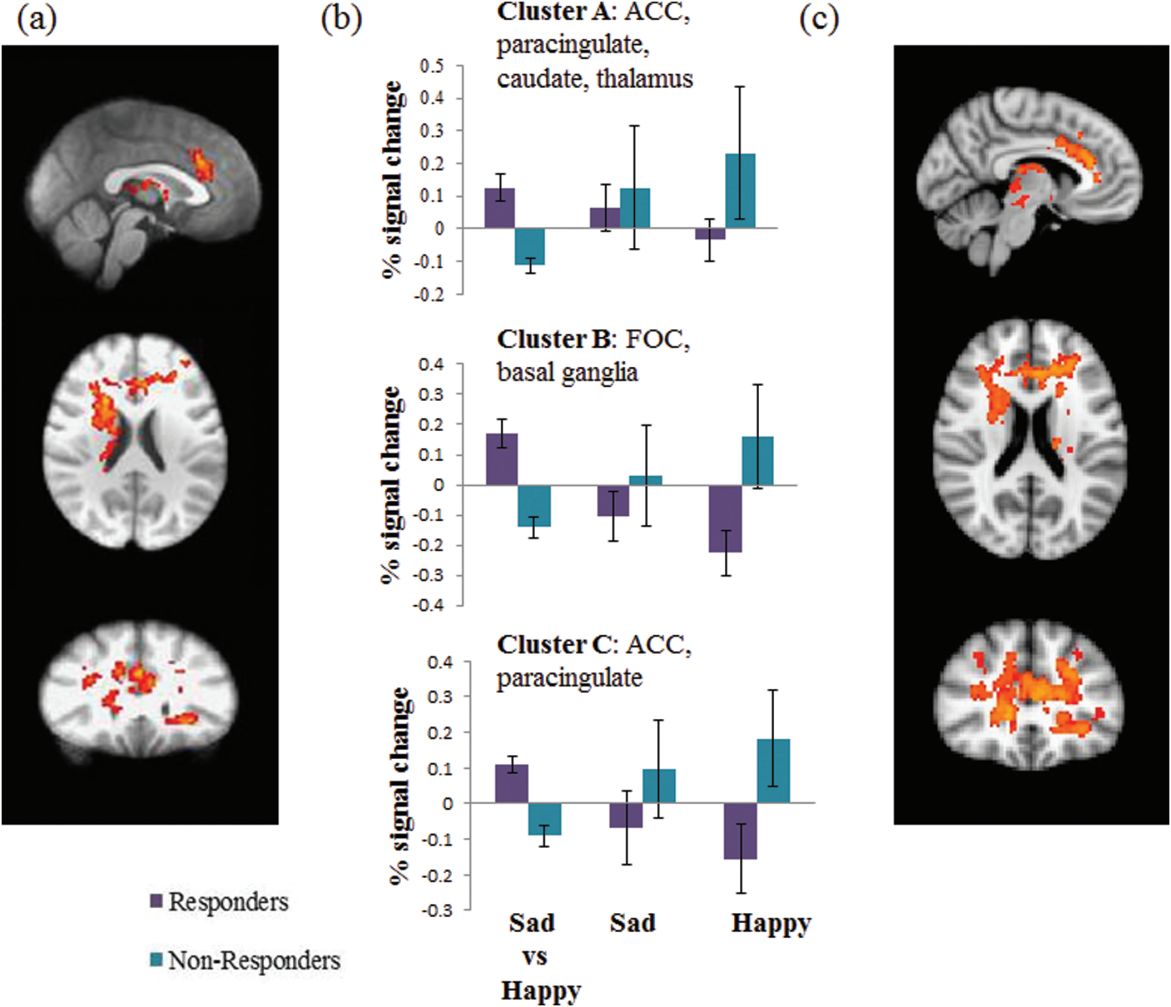
Baseline differences in neural response (percent signal change) at the whole-brain level in response to sad vs happy facial expressions differentiated between responders and nonresponders to 6 weeks of treatment with escitalopram. The figure represents (a) results of the exploratory analysis at the whole-brain level using a parametric approach (Gaussian Random Field Theory); (b) extracted signal change in the identified clusters (mean and SE); (c) results of the exploratory analysis at the whole-brain level using a nonparametric approach (Threshold-Free Cluster Enhancement). Details of the clusters can be found in Table 2. Analysis was thresholded at Z=2.3 and cluster-corrected with a FWE *P*<.05. ACC, anterior cingulate cortex; FOC fronto-orbital cortex; FWE, family wise error. Baseline 17-item Hamilton Depression Rating Scale (HAM-D) scores were entered as a covariate.

**Whole-Brain Analysis—**The exploratory analysis at the whole-brain level using a parametric approach revealed a greater activation to sad vs happy faces in treatment responders compared with nonresponders across a network of structures including ACC, paracingulate, right and left caudate, right thalamus and a small part of left thalamus, left putamen, and a small portion of fronto-occipital cortex, both when controlling for baseline depression HAMD severity and not (*P*<.05, FWE corrected; Figure 2). For details on functional clusters, see Table 2. The Threshold-Free Cluster Enhancement nonparametric method revealed a similar increased activation in response to sad vs happy faces in treatment responders vs nonresponders across a network of structures, with the peak in the ACC and inclusing paracingulate gyrus, bilateral middle frontal gyrus, bilateral frontal orbital cortex, frontal pole, bilateral thalamus, and left insula.

**Table 2.** Prediction of Clinical Response after 6 Weeks of Escitalopram Treatment from Baseline Differences in Pretreatment Neural Response to Sad Compared with Happy Facial Expressions

Cluster content	Peak voxel MNI Coordinates			Cluster size, voxels	Z-value	*P* value
	x	y	Z			
Parametric approach (Gaussian Random Field Theory)						
Cluster A: L middle frontal gyrus, ACC, paracingulate gyrus, R caudate, R thalamus leaking into L thalamus	-28	32	28	2173	3.66	.000000238
Cluster A: local maxima	22	12	16		3.59	
	24	-28	26		3.59	
	22	16	22		3.54	
	30	26	24		3.53	
	-6	30	20		3.48	
Cluster B: Frontal orbital cortex, L putamen, L caudate, L accumbens	-32	34	-4	544	3.68	.0276
Cluster B: local maxima	-18	22	-4		3.37	
	-28	40	-4		3.24	
	-22	48	-8		3.04	
	-24	12	-18		3.03	
	-22	44	-8		3.00	
Cluster C: ACC, paracingulate gyrus	26	36	0	523	3.7	.0336
Cluster C: local maxima	16	36	-4		3.37	
	18	42	2		3.27	
	34	42	-2		3.04	
	20	26	10		3.03	
	20	42	-8		2.89	
Nonparametric approach (Threshold-Free Cluster Enhancement)						
Cluster: ACC, paracingulate gyrus, bilateral middle frontal gyrus, bilateral frontal orbital cortex, frontal pole, bilateral thalamus, left insula	-4	30	16	6617	4.99	<.05

Abbreviations: ACC, anterior cingulate cortex.

The table shows functional clusters identified by the exploratory analysis at the whole brain level. Please refer to Figure 1 for more details.

**Figure 3.**
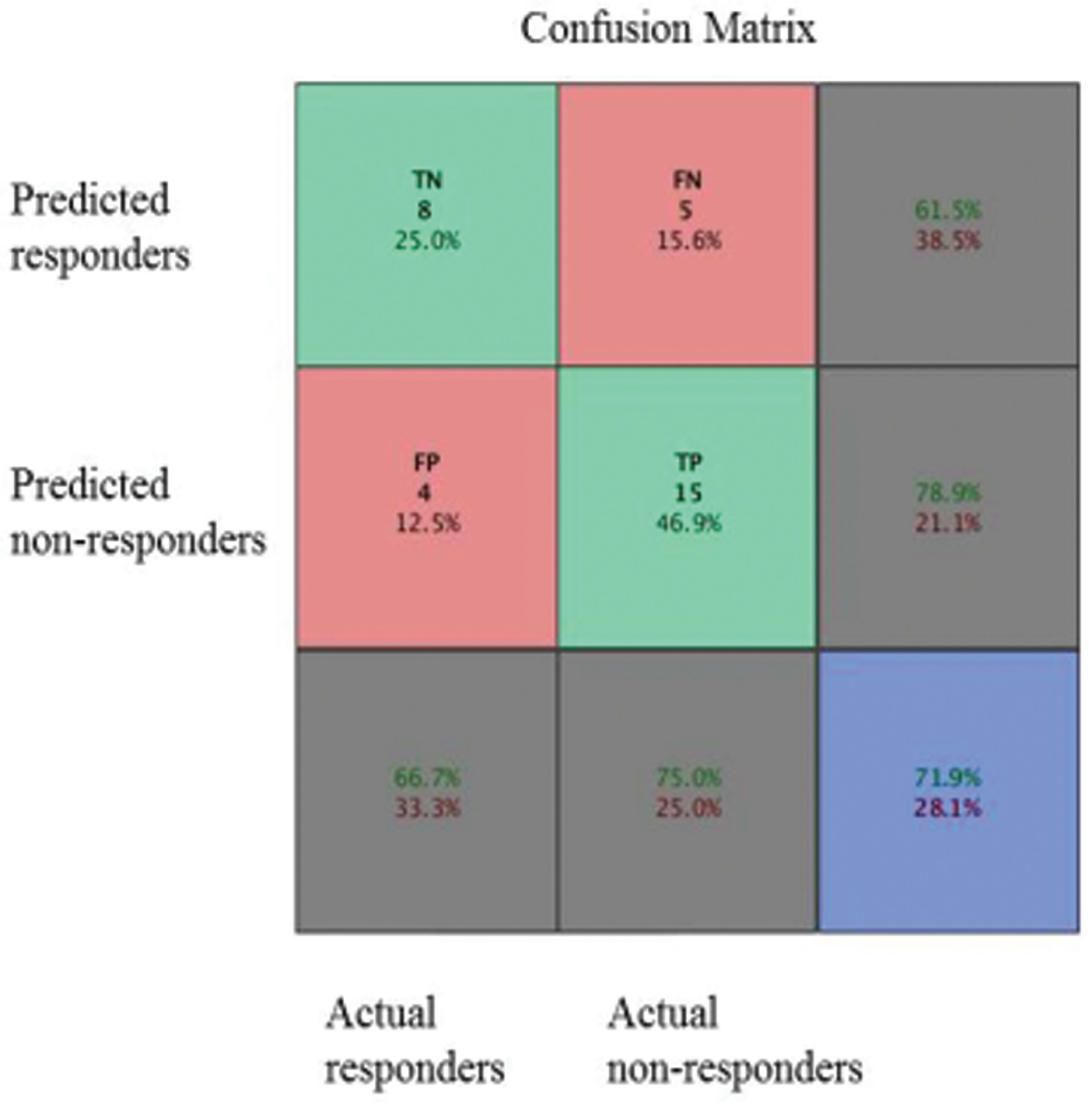
Confusion plot. Green squares represent correctly classified cases: TP, true positives; TN, true negatives, the number of correct classifications by the trained network, percentage of all cases they represent. Red squares represent incorrectly classified cases: FP, false positives, FN, false negatives, the number of correct classifications by the trained network, percentage of all cases they represent. The blue square represents the percentage of correct and incorrect classifications. The first row represents predicted nonresponders, of whom 61.5% were classified correctly and 38.5% incorrectly. The second row represents predicted responders, of whom 78.9% were classified correctly and 21.1% incorrectly. Of 12 nonresponders, 66.7% were correctly predicted as nonresponders and 33.3% were predicted as responders. Of 20 responders, 75% were correctly classified as responders and 25% were classified as nonresponders. Overall, 71.9% of the predictions were correct and 28.1% cases were classified incorrectly.

**Figure 4.**
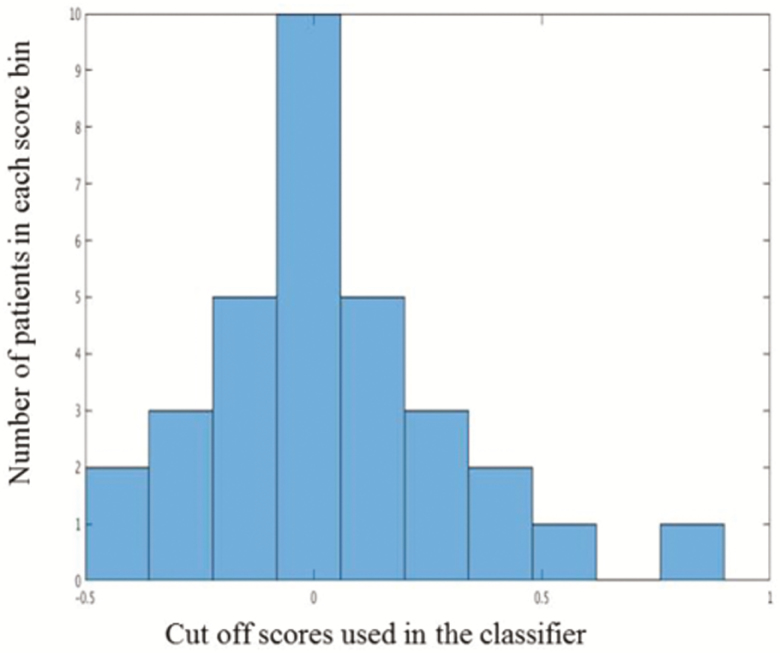
Histogram of the cut-off scores used in the classifier.

Prospective analysis allowed predicting classification into responders and nonresponders with moderate accuracy of 71.875%. The center of mass of the clusters is shown in Figure 2. A histogram of cut-off scores and confusion matrix are shown in Figures 3 and 4, respectively.

## Discussion

Our study showed that pretreatment neural activation of pgACC in response to subliminal emotional information was predictive of short-term (6 weeks) therapeutic response to an SSRI escitalopram in patients with depression. A similar pattern was observed in a network of cortical and limbic areas associated with depression. Our findings support the body of evidence pointing to the ACC as currently the most reliable marker of response to various modes of treatment for depression and show its role in response to emotional information presented below the level of conscious awareness.

Our findings are consistent with meta-analyses by Pizzagalli (2011) and Fu et al. (2013) and other more recent studies implicating the pgACC as the region in which baseline function is linked to future response to antidepressant treatment (e.g., Roy et al., 2010; Kozel et al., 2011; Miller et al., 2013; Victor et al., 2013; Dichter et al., 2015; Cullen et al., 2016; Vai et al., 2016; Crane et al., 2017; Klumpp et al., 2017). Of recent investigations, the study by Victor et al. (2013) was of particular relevance to our investigation because it also employed an implicit emotional task based on presentation of masked sad and happy faces. Victor et al. (2013) found a decrease in the haemodynamic response in the pgACC to the contrast of masked sad vs happy faces after 8 weeks of treatment with another SSRI, sertraline, in 10 participants with depression. They also found a positive correlation between symptomatic improvement in depression ratings and baseline pgACC activation to masked sad vs happy faces, implicating pgACC in antidepressant response and its prediction. Studying a larger group of participants and using a similar task based on masked presentation of emotions, we were also able to show differential baseline pretreatment activity of the pgACC in treatment responders and nonresponders.

The pgACC is a part of an extended medial prefrontal network, uniquely positioned with connections both to the amygdala and PFC (Drevets 2001, Mayberg 2003). It plays an important role in emotion appraisal and regulation, evaluation of the salience of emotional stimuli, and emotion-related learning (Stevens et al. 2011), the processes that are dysregulated in depression and improve with successful treatment. Since the seminal publication by Mayberg et al. (1997), a wide range of research has consistently implicated pgACC as a brain region linked to treatment response.

Increased activity in the pgACC was typically found to be predictive of better treatment outcomes regardless of the modality used to measure ACC activity (fMRI, positron emission tomography (PET), Magnetoencephalography, and Electroencephalography), design (i.e., resting state or task-based), and form of treatment employed (pharmacotherapy, transcranial magnetic stimulation, CBT, and sleep deprivation), as reviewed by Pizzagalli in 2011. Although only 5 of 23 studies included in this review were fMRI studies, other fMRI studies have since been published, including a meta-analysis by Fu et al. (2013) of 20 functional neuroimaging papers (14 fMRI studies, 10 using various emotional processing tasks, 4 using nonemotional tasks, and 6 PET studies in resting state conditions). Similar to Pizzagalli’s paper (2011), this meta-analysis showed a higher likelihood of improvement in response both to pharmacotherapy (14 included studies) or CBT (6 included papers) in patients with increased pretreatment activation in the pgACC as well as in subgenual and medial prefrontal cortices.

Since the publication of these meta-analyses, other studies, including ours (Godlewska et al., 2016), have added to the relatively consistent body of evidence suggesting increased ACC activation during emotional processing and resting state as a putative marker of good clinical response to treatment. Although the results are not unequivocal (Fu et al., 2013) and are still some way off routine clinical application, currently the pgACC is one of the most, if not the most, promising pretreatment imaging biomarkers of antidepressant response, because of both its postulated role in the pathophysiology of depression and the consistency of reports on its role in treatment response prediction.

In addition to the pgACC, our exploratory whole-brain analysis revealed a number of other regions showing a similar neural response to the employed task, that is, increased activity in response to sad vs happy facial expressions allowed differentiation between future treatment responders and nonresponders. These regions included paracingulate cortex, thalamus, caudate, putamen, nucleus accumbens, and orbitofrontal cortex (Figure 2). These structures are part of networks involved in the processing of emotionally valenced information and reward, and their disruption has been hypothesized to contribute to low mood and anhedonia, core symptoms of depression (Graham et al., 2013; Fettes et al. 2017).

The basal ganglia regions identified in this study (caudate nucleus, putamen, and nucleus accumbens) are involved in reward processing and form connections with the ACC and thalamus, with the ACC-basal ganglia-thalamus loop suggested to monitor unexpected events and to recruit adaptive mechanisms as required (Maia and Frank, 2011). Our results suggest that the pgACC is clearly a node in a distributed network of regions involved in processing of emotionally salient information. It is likely that differences in the function of this network, rather than solely in the pgACC function, are associated with antidepressant response.

The period of time when response to antidepressants was assessed, 6 weeks into treatment, was chosen to be consistent with common clinical practice. Six weeks is around the time when clinicians often make decisions as to whether to continue treatment unchanged, adjust the dose, or switch to a different medication, and is in line with current treatment guidelines (NICE Pathways, 2017). A reliable marker of this early response could potentially save a patient from unnecessary weeks of delay in finding treatment they respond to. Treatment response was defined as baseline at least 50% decrease in HAMD scores as this definition, although somehow arbitrary, is a commonly used measure (Angst et al., 1993).

To be clinically useful, a biomarker of treatment response needs to classify patients into responders and nonresponders with reasonable accuracy; so far, no known biomarker has been consistently replicated in subsequent studies with accuracy high enough to be deemed useful in clinical practice (Fu and Costafreda, 2013). Predictive analysis performed in this study allowed classification of participants into responders and nonresponders with an accuracy of 71.9%, which differs from a probability of 0.5 with a *P* of .02. However, caution is required with this estimate of accuracy as LOO approaches to validation, as used in the current study, will tend to overestimate classifier accuracy compared with validation in a fully held-out sample (Hastie et al., 2009). This limitation is related to a number of statistical and methodological aspects of LOO procedures. First, clinically relevant classification requires between-dataset generalization of classification performance (predictions need to be made on completely unseen data), which is not accounted for by LOO procedures based only on within-dataset performance. Second, individual data points in LOO procedures will be used in all but one training sets, meaning that influential (e.g., outlying) data points can have exaggerated effects on classifier performance across training sets, which can skew estimates of classifier performance. As a result, it will be essential to test whether pgACC activity is able to meaningfully predict treatment response in a fully held-out sample of patients.

A number of studies have used machine learning approaches to derive classifiers from fMRI data. These approaches tend to have many more predictors (i.e., voxels) than data points (patients) and are also difficult to interpret from a mechanistic perspective as they incorporate complex interactions between predictors. In our study, we used a very simple, univariate outcome from a prespecified region, which provides a mechanistically transparent predictor. Clearly, however, it may be possible to improve on the predictive performance reported here by employing a multivariate approach to combine different features in the classifier. Additionally, there may well be alternative methods for deriving the predictive features from the fMRI data. In the current study, we used mean signal change from clusters, defined in the training set. The clusters were defined on the basis of anatomical location (within the ACC) and statistical significance. It may be that using alternative methods for cluster/feature definition (e.g., changing the level of statistical significance for the cluster or relaxing the anatomical specification) would increase the information contained in the cluster and improve classification performance.

The ACC response seems to predict positive therapeutic outcome to many different kinds of treatment (Pizzagalli, 2011; Fu et al., 2013). Therefore, it does not currently point to selection of a particular antidepressant treatment modality or psychotherapy in preference to pharmacotherapy. Equally, it does not suggest an alternative treatment regime for patients with low ACC responsivity who, at the moment, are predicted to do less well with various kinds of antidepressant therapies. At the same time, it may play an important role in identifying people with poor prognosis who can be given more intensive treatment from the start, leading to improving overall time to remission. It might also serve as a marker of efficacy when testing new drugs for antidepressant properties.

Our study has some limitations. The main limitation is the small size of the group. The group itself was composed of carefully chosen drug-free patients, yet increasing the number would increase the power and allow for more complex analyses combining different putative markers, an approach aiming at increasing accuracy of classification. The lack of a control group may be considered as another limitation; however, the aim of the current study was to explore markers of treatment response prediction, for which a group of healthy volunteers or placebo-treated patients is not strictly necessary. One limitation reflects a general question of feasibility of using imaging biomarkers in clinical settings, as scanning is not yet widely available and the procedure is still relatively costly. However, if the prediction using imaging biomarkers becomes sufficiently accurate, benefits including a decrease of depression burden on both individuals and society achieved through more efficient therapeutic processes could make it cost effective.

In summary, our study has shown that pretreatment pgACC activity is predictive of response to antidepressant treatment after 6 weeks. It has also identified other brain regions where differential activity in response to an implicit emotional task had a similar predictive value. Our study adds to the growing body of evidence pointing at the pgACC as a reliable predictor of subsequent treatment response to a variety of therapeutic approaches to depression. Although the accuracy of classification in our study was moderate, it was higher than by chance. Given that the pgACC pretreatment function as response prediction marker has been the most consistently replicated neuroimaging finding, it makes it the most promising putative fMRI-based treatment response biomarker. To understand better the potential of pgACC imaging in this context, future studies are needed, on large groups and in patients at different stages of depression, employing machine learning approaches to combine pgACC effect with other neuroimaging and/or behavioral measures to increase classification accuracy.

## Funding

This work was supported by Medical Research Council (grant no. MR/K022202) and by the NIHR Oxford Health Biomedical Research Centre. The views expressed are those of the authors and not necessarily those of the NHS, the NIHR, or the Department of Health.

## Interest Statement

C.J.H. has received consultancy fees from P1vital, Servier, Lundbeck, and J&J. She also had grant income from UCB and J&J. M.B. is employed on a part-time basis by P1vital ltd. He has received travel expenses from Lundbeck and acted as a consultant for J&J. B.R.G. has received travel expenses from Janssen UK. A.I. and R.N. have no conflict of interest to declare.
